# The first two complete mitochondrial genomes for the family Triglidae and implications for the higher phylogeny of Scorpaeniformes

**DOI:** 10.1038/s41598-017-01654-y

**Published:** 2017-05-08

**Authors:** Lei Cui, Yuelei Dong, Fenghua Liu, Xingchen Gao, Hua Zhang, Li Li, Jingyi Cen, Songhui Lu

**Affiliations:** 1 Key Laboratory of Eutrophication and Red Tide Prevention, Research Center for Harmful Algae and Marine Biology, Jinan University, Guangzhou, 510632 China; 2 Chinese Sturgeon Research Institute, Three Gorges Corporation, Yichang, 443100 China

## Abstract

The mitochondrial genome (mitogenome) can provide useful information for analyzing phylogeny and molecular evolution. Scorpaeniformes is one of the most diverse teleostean orders and has great commercial importance. To develop mitogenome data for this important group, we determined the complete mitogenomes of two gurnards *Chelidonichthys kumu* and *Lepidotrigla microptera* of Triglidae within Scorpaeniformes for the first time. The mitogenomes are 16,495 bp long in *C. kumu* and 16,610 bp long in *L. microptera*. Both the mitogenomes contain 13 protein-coding genes (PCGs), 2 ribosomal RNA (rRNA) genes, 22 transfer RNA (tRNA) genes and two non-coding regions. All PCGs are initiated by ATG codons, except for the cytochrome coxidase subunit 1 (*cox1*) gene. All of the tRNA genes could be folded into typical cloverleaf secondary structures, with the exception of tRNA^Ser(AGN)^ lacks a dihydrouracil (DHU) stem. The control regions are both 838 bp and contain several features common to Scorpaeniformes. The phylogenetic relationships of 33 fish mitogenomes using Bayesian Inference (BI) and Maximum Likelihood (ML) based on nucleotide and amino acid sequences of 13 PCGs indicated that the mitogenome sequences could be useful in resolving higher-level relationship of Scorpaeniformes. The results may provide more insight into the mitogenome evolution of teleostean species.

## Introduction

Generally, the fish mitogenome is a circular and double-stranded molecule ranging from 15 to 19 kilobases in length. It usually contains two ribosomal RNA genes (12S rRNA and 16S rRNA), 22 transfer RNA genes (tRNAs), 13 protein-coding genes (PCGs) and two typical non-coding control regions (control region (CR) and origin of the light strand (O_L_)) with regulatory elements essential for transcription and replication^1, 2^. Because of the characteristics of coding content conservation, maternal inheritance, rapid evolution, and low levels of intermolecular genetic recombination, mitogenomes have become increasingly effective and popular markers for molecular research, such as phylogenetic molecular evolution, population genetics, phylogenetics, and comparative and evolutionary genomics^3, 4^. In addition to comparing nucleotide and amino acid sequence applying to molecular evolution, the complete mitogenome of tRNA secondary structure, gene rearrangement and models of the control of replication and transcription have been used extensively for deep-level phylogenetic inference in taxonomy in recent decades^5, 6^.

Scorpaeniformes, whose members are known for their commercial importance (e.g., *Sebastes*, *Ophiodon*) and venomous spines (e.g., *Pterois, Scorpaena*), is one of the largest and most morphologically diverse teleostean orders of fish, with more than 1400 species classified in 38 families depending on the taxonomy^7, 8^. Due to commercial overfishing^9^, four scorpionfishes have been protected by the International Union for Conservation of Nature (IUCN). In addition, some of Scorpaeniform fishes, such as Synanceiidae (stonefishes), have caused numerous human injuries due to their venom^10–12^. Several partial mitochondrial gene sequences of the 12S rRNA, the 16S rRNA and the complete tRNA-Val and nuclear genes of the large ribosomal subunit (28S), histone H3, and TMO-4c4 from Scorpaeniform have been sequenced and extensively used for phylogenetic analysis^13^. Nevertheless, the short genes do not provide enough information for phylogenetic relationships and sometimes result in controversial signals^14, 15^. Triglidae fishes belong to a moderately large family of the order Scorpaeniform, comprising approximately 14 genera and 100 species in tropical and temperate waters of the world’s oceans^16^. Because of medical importance and commercial value, many researchers have studied some aspects of Triglidae’s ecological distribution, morphology and biological habits^17–19^, but few investigations have focused on the taxonomy based on molecular features.

To date, more than 100 complete mitogenomes from teleostean species have been sequenced, but only 28 species in 5 families from Scorpaeniform are available in GenBank, which does not include the family Triglidae. The gurnards *Chelidonichthys kumu* and *Lepidotrigla microptera* are two species of the family Triglidae and are distributed in New Zealand, Australia, Japan, Malaysia, South Korea and China. However, the complete mitogenomes of *C. kumu* and *L. microptera* have not been determined. To understand the higher-level relationships of Scorpaeniformes, in this study, we sequenced the complete mitochondrial genome of the two Triglidae species and investigated the gene content and organization compared with other species. Furthermore, we reconstructed a phylogenetic tree based on PCG sequences for the purpose of analyzing the evolutionary relationships among Scorpaeniformes fish. In addition, the characterization of the *C. kumu* and *L. microptera* mitogenomes may provide more insight into the genesis and mitogenome evolution of teleostean species.

## Methods

### Sampling and DNA extraction

The specimens of *C. kumu* and *L. microptera*, which did not involve endangered or protected species according to the IUCN Red List, were collected in the Pearl River estuary (N 21°57′, E 133°47′), China, in July 2016. Our study was conducted with the approval from the Institutional Animal Care and Use Committee at Jinan University. All operations were performed according to international guidelines concerning the care and treatment of experimental animals. All samples were preserved in 95% ethanol and were stored at −80 °C until DNA extraction. Total genomic DNA was extracted from dorsal muscle tissue samples using the Animal Tissue Genomic DNA Extraction Kit (SangonBiotech, China). Extracted DNA was used to amplify the complete mitogenomes by PCR.

### PCR amplification and sequencing

For the amplification of the *C. kumu* and *L. microptera* mitogenomes, several primer pairs were designed based on the aligned mitogenome sequences of *Scalicus amiscus* (GenBank: AP004441.1) (Table S1)^20^. The PCR amplifications were performed with LA Taq DNA polymerase using Premix LA Taq (Takara, China) under the following conditions: an initial denaturation step at 95 °C for 3 min, followed by 35 cycles of denaturation at 95 °C for 30 s, annealing at 50 °C for 30 s and elongation at 72 °C for 1–5 min. All the PCR products were sent to Beijing Genomics Institute and sequenced using the primer walking method with a 3730XL DNA Analyzer. The obtained sequences had 100% coverage of the PCR products.

### Sequence analysis

The sequences were manually checked and assembled with the program Seqman within Lasergene software. The final complete sequence annotation was performed using NCBI BLAST (http://blast.ncbi.nlm.nih.gov/Blast) and the DNAStar package (DNAStar Inc. Madison, WI, USA). The location of the two rRNAs and the 13 PCGs for each species were primarily identified through Dual Organellar Genome Annotator (DOGMA)^21^. The majority of the transfer RNA (tRNA) genes were identified by the tRNA-scan-SE1.21 from the website http://lowelab.ucsc.edu/tRNAscan-SE/ using the default search mode and the ‘Mito/chloroplast’ source^22^. To infer the secondary structures of tRNA molecules, we used a commonly accepted comparative approach to correct for unusual pairings with RNA-editing mechanisms that are well known in fish mitogenomes. The software RNAstructure was used to draw the secondary structure of tRNA genes and the origin of light strand replication (O_L_)^23^. The codon usage of the 13 PCGs was calculated using MEGA 6^24^. Tandem repeats in the control region (CR) were analyzed using the Tandem Repeats Finder program (http://tandem.bu.edu/trf/trf.html)^25^. The nucleotide composition skewness was measured according to the following formulas: AT skew [(A − T)/(A + T)] and GC skew [(G − C)/(G + C)]^26^.

The complete mitochondrial genomic DNA sequence of the *C. kumu* and the *L. microptera* were deposited into the GenBank database under the accession numbers KY379222 and KY012348, respectively.

### Phylogenetic analysis

To investigate the phylogenetic relationships among fish, a total of 27 Scorpaeniformes mitogenomes available in GenBank were used (Table 1). The mitogenomes of Perciformes fish (*Caesio cuning*, *Emmelichthys struhsakeri* and *Banjos banjos*) were used as outgroups. The amino acid sequences and nucleotide sequence for each species of the 13 PCGs were aligned using default settings and concatenated, which were used for phylogenetic analysis via the Maximum Likelihood (ML) and Bayesian inference (BI) methods using raxmlGUI and MrBayes v 3.2.4, respectively^27, 28^. Every gene was aligned separately using Clustal X with default settings^29^. GTR+ I+ G was selected as the appropriate model for the nucleotide sequences by Modeltest 3.7 based on Akaike’s information criterion (AIC)^30^. MtArt+ I+ G+ F was the appropriate model for the amino acid sequence dataset according to ProtTest 3.4 based on AIC^31^. Four independent runs were allowed to run simultaneously for 1,000,000 generations and each was sampled every 1,000 generations, with the first 25% discarded as burn-in. Stationarity was considered to be reached when the average standard deviation of split frequencies was much less than 0.01. The resulting phylogenetic trees were drawn in FigTree v1.4.3.

**Table 1:** List of the complete mitogenomes of Scorpaeniformes fish.

Family	Species	Accession number	Size (bp)	Whole genome composition							PCGs	
				A%	G%	T%	C%	A + T%	AT skew	GC skew	AT skew	GC skew
Cottidae	*Icelus spatula*	KT004432	16384	26.43%	17.43%	30.04%	26.03%	52.46%	0.0078	−0.2656	−0.0995	−0.2938
Cottidae	*Cottus szanaga*	KX762050	16518	26.51%	17.39%	29.94%	26.16%	52.67%	0.0067	−0.2650	−0.0863	−0.3002
Cottidae	*Trachidermus fasciatus*	JX017305	16536	26.33%	18.13%	30.07%	25.47%	51.80%	0.0167	−0.2478	−0.0819	−0.2755
Cottidae	*Cottus poecilopus*	EU332750	16560	25.69%	18.18%	30.39%	25.74%	51.43%	−0.0011	−0.2513	−0.1047	−0.2825
Cottidae	*Cottus dzungaricus*	NC_024739	16527	26.93%	17.07%	29.70%	26.30%	53.23%	0.0119	−0.2701	−0.0824	−0.3137
Cottidae	*Cottus hangiongensis*	NC_014851	16598	25.48%	18.22%	30.40%	25.87%	51.36%	−0.0075	−0.2506	−0.1140	−0.2847
Cottidae	*Enophrys diceraus*	NC_022147	16976	27.53%	16.65%	28.64%	27.19%	54.71%	0.0062	−0.2648	−0.0876	−0.2990
Hexagrammidae	*Hexagrammos lagocephalus*	KP682334	16505	26.98%	17.26%	29.48%	26.29%	53.27%	0.0130	−0.2615	−0.0844	−0.3004
Hexagrammidae	*Hexagrammos otakii*	KR362879	16513	26.60%	17.33%	29.87%	25.90%	52.80%	0.0189	−0.2656	−0.0732	−0.3071
Hexagrammidae	*Hexagrammos agrammus*	AB763992	16514	26.88%	17.23%	29.72%	26.15%	53.03%	0.0137	−0.2659	−0.0845	−0.3012
Peristediidae	*Satyrichthys amiscus*	AP004441	16526	27.06%	17.00%	28.56%	27.38%	54.44%	−0.0058	−0.2537	−0.1032	−0.2890
Scorpaenidae	*Scorpaenopsis cirrosa*	NC_027735	16966	27.91%	17.71%	28.02%	26.35%	54.27%	0.0288	−0.2254	−0.0607	−0.2448
Sebastidae	*Sebastes oblongus*	NC_024549	16396	27.91%	16.96%	28.72%	26.41%	54.32%	0.0276	−0.2574	−0.0601	−0.2842
Sebastidae	*Sebastes thompsoni*	NC_027447	16405	27.98%	17.04%	28.21%	26.77%	54.75%	0.0222	−0.2468	−0.0681	−0.2783
Sebastidae	*Sebastes minor*	NC_027444	16408	27.79%	17.33%	27.63%	27.25%	55.04%	0.0099	−0.2292	−0.0821	−0.2611
Sebastidae	*Sebastes trivittatus*	NC_027446	16409	27.86%	17.09%	28.37%	26.67%	54.54%	0.0218	−0.2480	−0.0695	−0.2791
Sebastidae	*Sebastes pachycephalus*	KF836442	16415	27.77%	17.24%	28.66%	26.33%	54.10%	0.0266	−0.2488	−0.0640	−0.2788
Sebastidae	*Sebastes longispinis*	KJ834061	16445	27.91%	17.12%	28.31%	26.66%	54.57%	0.0230	−0.2462	−0.0704	−0.2716
Sebastidae	*Sebastes steindachneri*	NC_027445	16450	27.36%	17.54%	28.01%	27.09%	54.46%	0.0049	−0.2298	−0.0879	−0.2590
Sebastidae	*Sebastes taczanowskii*	KJ525744	16452	27.71%	17.29%	28.53%	26.47%	54.18%	0.0229	−0.2452	−0.0714	−0.2750
Sebastidae	*Sebastes hubbsi*	KJ525745	16453	27.86%	17.20%	28.28%	26.66%	54.52%	0.0221	−0.2436	−0.0675	−0.2716
Sebastidae	*Sebastes vulpes*	NC_027438	16462	27.71%	17.14%	28.61%	26.55%	54.25%	0.0214	−0.2506	−0.0713	−0.2811
Sebastidae	*Sebastes owstoni*	KJ834063	16465	27.71%	17.30%	28.41%	26.57%	54.28%	0.0210	−0.2430	−0.0753	−0.2726
Sebastidae	*Sebastes inermis*	KF725093	16504	27.77%	17.13%	28.34%	26.76%	54.53%	0.0186	−0.2466	−0.0719	−0.2737
Sebastidae	*Sebates schlegeli*	AY491978	16525	27.47%	17.45%	28.74%	26.34%	53.80%	0.0210	−0.2444	−0.0698	−0.2746
Sebastidae	*Sebastiscus marmoratus*	GU452728	17301	28.69%	16.51%	28.14%	26.67%	55.36%	0.0364	−0.2605	−0.0606	−0.2855
Synanceiidae	*Synanceia verrucosa*	NC_026989	16506	31.01%	15.06%	25.60%	28.34%	59.35%	0.0451	−0.2593	−0.0292	−0.2883
Triglidae	*Chelidonichthys kumu*	KY379222	16495	26.63%	17.04%	31.13%	25.20%	51.83%	0.0277	−0.2925	−0.0260	−0.3475
Triglidae	*Lepidotrigla microptera*	KY012348	16610	26.53%	17.20%	31.22%	25.05%	51.58%	0.0287	−0.2895	−0.0188	−0.3647

Complete mitogenomes and PCGs composition bias of all Scorpaeniformes fish mitogenomes available from GenBank. The accession numbers and the size of mitogenomes are shown.

## Results and Discussion

### Genome organization and structure

**Figure 1:**
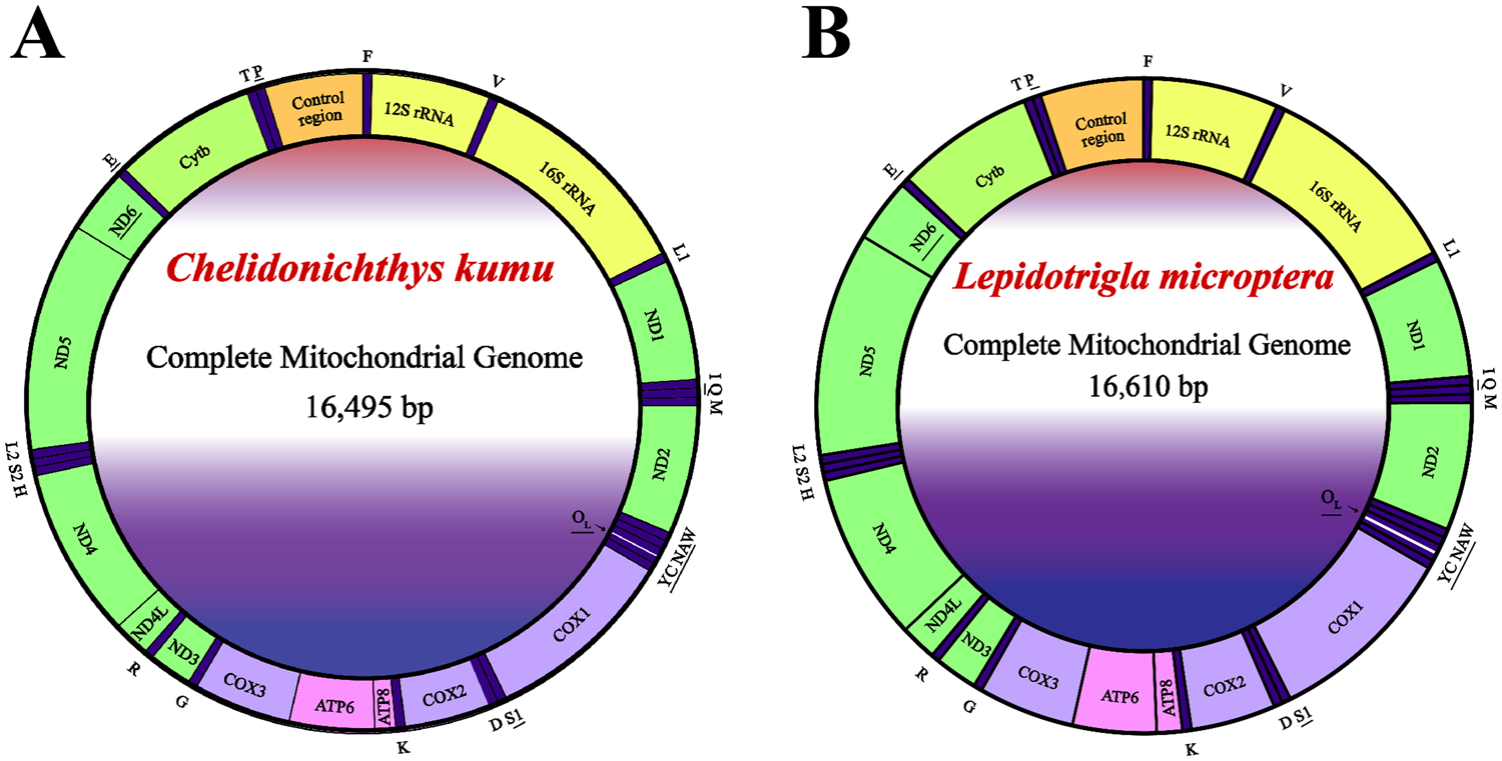
Circular map of the mitogenome of *C. kumu* (**A**) and *L. microptera* (**B**). Transfer RNAs are designated by the IUPAC-IUB single letter amino acid codes (L1: trnL^CUN^; L2: trnL^UUR^; S1: trnL^AGN^; S2: trnL^UCN^) Genome organization names not underlined indicate coding sequence on the heavy strand and those with underline indicate coding sequence on the light strand.

The complete mitochondrial genome sequences of *C. kumu* and *L. microptera* are 16,495 bp and 16,610 bp in length. Both of them are closed circular molecules. The two mitogenomes are typical of other Scorpaeniformes fish mitogenomes, including 13 PCGs (*cox1–3, nad1–6, nad4L, atp6, atp8 and cob*), 2 rRNA genes (12S rRNA and 16S rRNA), 22 transfer RNA genes (one for each amino acid and two each for serine and leucine) and two non-coding regions (O_L_ and the control region (CR)) (Fig. 1). Among these 37 genes, twenty-three genes are transcribed on the heavy strand (H-strand), except for ND6 and eight tRNA genes (Gln, Ala, Asn, Cys, Tyr, Ser (UCN), Glu, and Pro). The order and orientation of genes in the *C. kumu* and *L. microptera* mitogenomes are identical to the other Scorpaeniformes species sequenced to date^20, 32, 33^.

### Skewness, overlapping, and intergenic spacer regions

The mitogenome of *C. kumu* has a 48 bp overlap between genes in ten locations ranging from 1 to 20 bp, with the longest 20 bp overlap located between 16S RNA and trnL. In seven locations of the *L. microptera* mitogenome, genes overlapped by 1 to 50 bp, and the longest 50 bp overlap is located between ATPase 8 and ATPase 6. The mitogenomes of *C. kumu* contains 65 bp of intergenic spacer sequences (1 to 36 bp in length) spread over 11 regions, while there are 78 bp nucleotides dispersed in 10 intergenic spacers, ranging in size from 1 to 38 bp, in the mitogenomes of *L. microptera*. The longest spacer sequences in the two species are located between the trnN and the trnC, which are extremely C + G rich. The nucleotide compositions of the H-strand of *C. kumu* and *L. microptera* mitogenomes are as follows: A = 4,393(26.6%), T = 4,156 (25.2%), G = 2,811 (17.0%), and C = 5,135 (31.1%); A = 4,407(26.5%), T = 4,161 (25.1%), G = 2,857 (17.2%), and C = 5,185 (31.2%), respectively. The total nucleotide composition of the *C. kumu* and *L. microptera* mitogenomes are 51.8% and 51.6% A + T rich, respectively. Compared with other Scorpaeniformes species, the enrichments are lower than many species sequenced, i.e. *I. spatula* (52.5%), *H. lagocephalus* (53.2%), *C. szanaga* (52.7%), *C. dzungaricus* (53.2%), *S. amiscus* (54.4%), *S. marmoratus* (55.3%) and *S. verrucosa* (59.3%). In contrast, these enrichments are slightly higher compared with those of *C. poecilopus* (51.4%) and *Cottus hangiongensis* (51.3%). Both of the highest A + T contents (60.9% and 59.8%) were detected in the control region, which is consistent with previous research on other fish^20, 34^. Additionally, the AT skew (0.0277) for the *C. kumu* mitogenome is slightly positive, indicating a higher occurrence of As to Ts, and its GC skew (−0.2925) is negative, indicating a higher content of Cs than Gs. The AT skew (0.0287) and GC skew (−0.2895) for the *L. microptera* mitogenome are identical to those of the *L. microptera* mitogenome. These findings are similar to all sequenced Scorpaeniformes mitogenomes to date, except for *Cottus poecilopus*, *Cottus hangiongensis* and *Satyrichthys amiscus* ^20, 34^, which have a negative AT skew.

### Protein-coding genes

**Figure 2:**
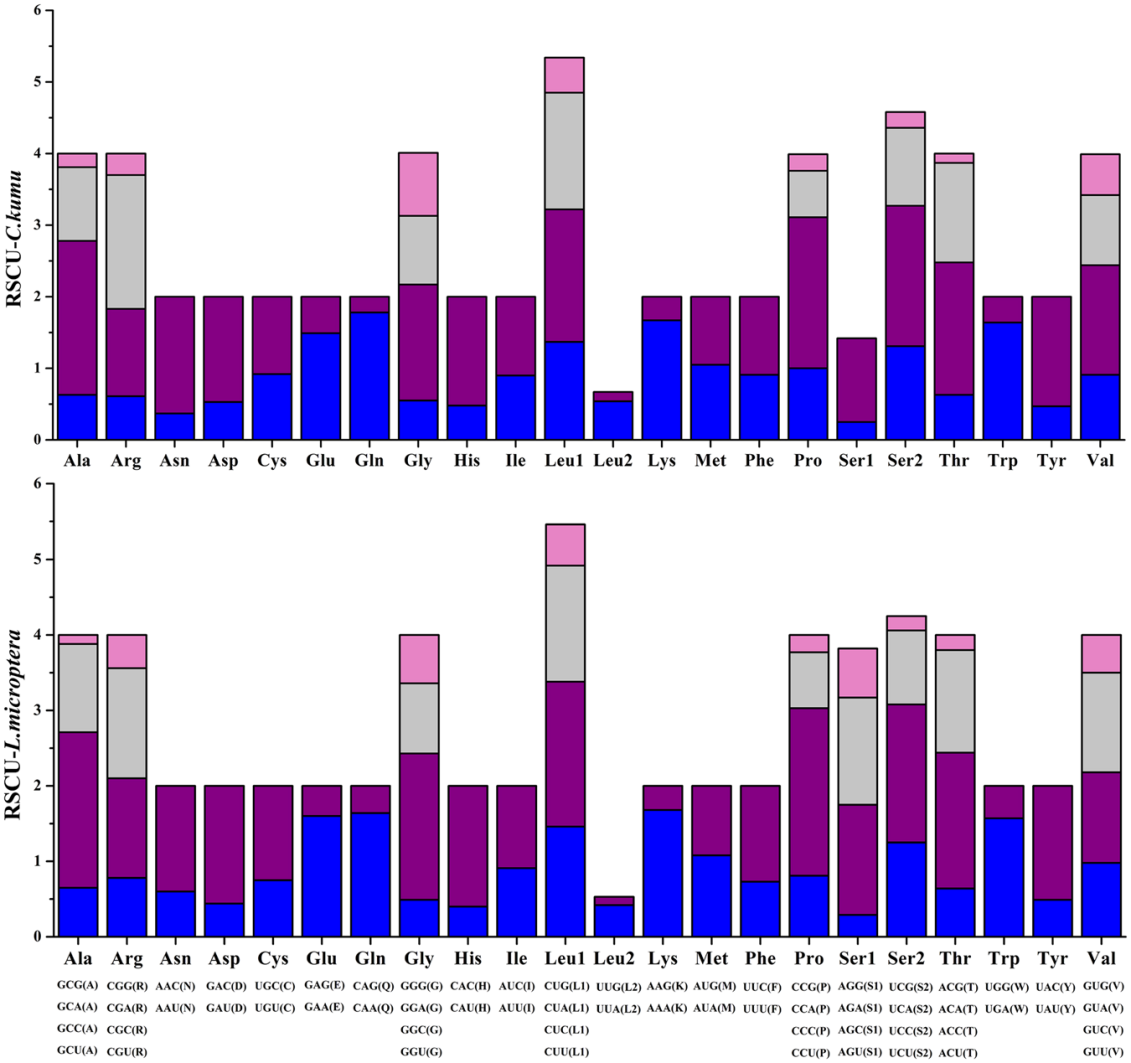
The relative synonymous codon usage (RSCU) in the mitogenomes of *C. kumu* and *L. microptera*.

**Figure 3:**
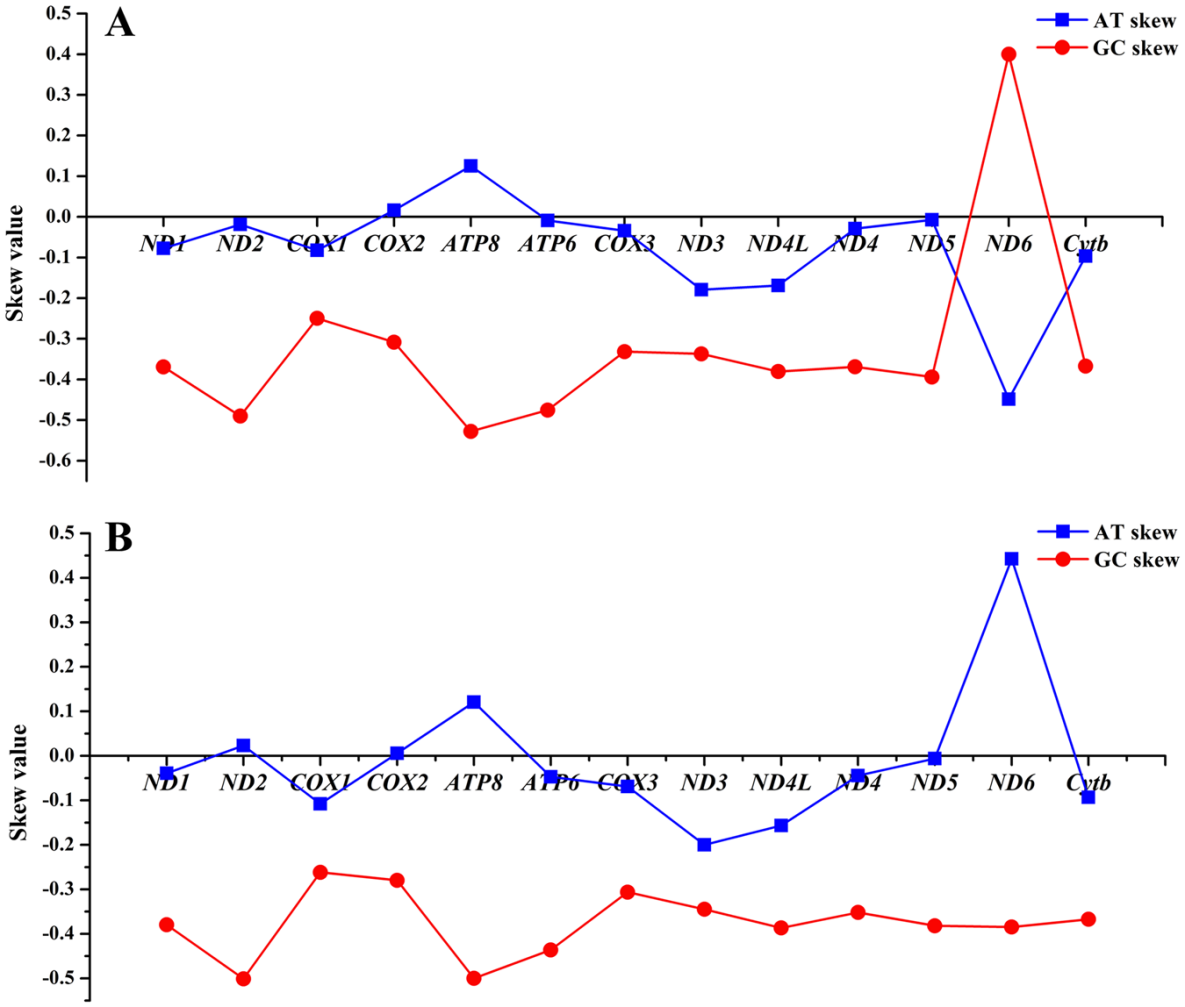
Graphical illustration showing the AT and GC skew in the PCGs of the mitochondrial genome of *C. kumu* (**A**) and *L*. *microptera* (**B**).

The total nucleotide length of the 13 PCGs in *C. kumu* and *L. microptera* are 11,429 bp and 10,450 bp, respectively. The start and stop codons of the 13 PCGs in the *C. kumu* and *L. microptera* mitogenomes are shown in Table 2. Methionine (ATG) is the start codon for most PCGs genes, except for *cox1*, which utilizes GTG, an accepted canonical mitochondrial start codon for vertebrate mitogenomes^35–37^. The stop codons (TAA, TTA and TAG) and one incomplete stop codon (T) are utilized in these PCGs. In *C. kumu*, *ND2* ends with TTA, *ND3* ends with TAG, three genes (cox2 *ND4* and *cytb*) use T and the remaining genes terminate with TAA. The stop codons of the two fish mitogenomes are identical, with the exception of T and TAG as stop codons for *ATP8* and *ND5*, respectively, in *L. microptera*. The incomplete stop codon is usually found in metazoan mitogenomes, which is presumably completed via post-transcriptional polyadenylation^38^. Relative synonymous codon usage values for the *C. kumu* and *L. microptera* mitogenomes are summarized in Table S2 and Fig. 2. The total number of codons in PCGs of *C. kumu* is 3808, where AGA and AGG codons are not represented. In the *L. microptera* mitogenome, the 13 PCGs have full codons (total number: 3431). Leucine 1 (Leu 1, 586), alanine (Ala, 351), and threonine (Thr, 303) are the most common amino acids in *C. kumu* mitogenome PCGs. In *L. microptera* mitochondrial proteins, the three amino acids (Leu 1, 535; Ala, 297 and Thr, 294) are the most common. The A + T content of the 13 PCGs of *C. kumu* and *L. microptera* mitogenomes is 50.6% and 50.5%, respectively. Furthermore, the AT skew and GC skew values of the PCGs of the two species are shown in Fig. 3. The most AT skew is negative, except for five genes (*cox2*, *ATP8* for *C. kumu* and *ND2*, *cox2*, *ATP8* and *ND6* for *L. microptera*). All GC skew values are negative with the exception of the *ND6* gene of *C. kumu*, which had a positive GC skew. This result suggests that more Ts and Cs are present in most PCGs, which is consistent with most previous observations^34, 39^.

**Table 2:** Mitochondrial genome organization of *C. kumu* and in *L. microptera*.

Feature	Strand	*C. kumu*			*L. microptera*		
		Position	Spacer (+)/Overlap (−)	Start/Stop codon	Position	Spacer (+)/Overlap (−)	Start/Stop codon
tRNA-Phe (F)	H	1–68	0		1–68	0	
12S rRNA	H	69–1011	0		69–1109	0	
tRNA-Val (V)	H	1015–1086	3		1110–1181	0	
16S rRNA	H	1087–2799	0		1182–2876	0	
tRNA-Leu (L1)	H	2780–2854	−20		2877–2950	0	
ND1	H	2855–3829	0	ATG/TAA	2951–3925	0	ATG/TAA
tRNA-Ile (I)	H	3834–3903	4		3930–3999	4	
tRNA-Gln (Q)	L	3903–3973	−1		4000–4070	0	
tRNA-Met (M)	H	3973–4041	−1		4070–4138	−1	
ND2	H	4042–5087	0	ATG/TTA	4139–5185	0	ATG/TTA
tRNA-Trp (W)	H	5088–5158	0		5185–5255	−1	
tRNA-Ala (A)	L	5160–5228	1		5257–5325	1	
tRNA-Asn (N)	L	5230–5302	1		5327–5399	1	
O_L_	L	5303–5338	0		5400–5437	0	
tRNA-Cys (C)	L	5339–5405	36		5438–5502	38	
tRNA-Tyr (Y)	L	5406–5475	0		5503–5572	0	
COX1	H	5477–7027	1	GTG/TAA	5574–7124	1	GTG/TAA
tRNA-Ser (S1)	L	7028–7098	0		7125–7195	0	
tRNA-Asp (D)	H	7102–7174	3		7199–7271	3	
COX2	H	7181–7871	6	ATG/T	7292–7983	20	ATG/T
tRNA-Lys (K)	H	7872–7945	0		7984–8057	0	
ATPase 8	H	7947–8114	1	ATG/TAA	8059–8266	1	ATG/T
ATPase 6	H	8105–8788	−10	ATG/TAA	8217–8900	−50	ATG/TAA
COX3	H	8788–9570	−1	ATG/TAA	8900–9685	−1	ATG/TAA
tRNA-Gly (G)	H	9570–9641	−1		9685–9756	0	
ND3	H	9642–9992	0	ATG/TAG	9757–10107	0	ATG/TAG
tRNA-Arg (R)	H	9991–10059	−2		10106–10174	−2	
ND4L	H	10060–10356	0	ATG/TAA	10175–10471	0	ATG/TAA
ND4	H	10350–11731	−7	ATG/T	10465–11845	−7	ATG/T
tRNA-His (H)	H	11731–11799	−1		11846–11914	0	
tRNA-Ser (S1)	H	11800–11867	0		11915–11982	0	
tRNA-Leu (L1)	H	11872–11944	4		11987–12059	4	
ND5	H	11945–13783	0	ATG/TAA	12060–13898	0	ATG/TAG
ND6	L	13780–14301	−4	ATG/TAA	13895–14416	−4	ATG/TAA
tRNAGlu (E)	L	14302–14370	0		14417–14485	0	
Cytb	H	14376–15516	5	ATG/T	14491–15631	5	ATG/T
tRNA-Thr (T)	H	15517–15588	0		15632–15703	0	
tRNA-Pro (P)	L	15588–15657	−1		15703–15772	−1	
Control region	H	15658–16495	0		15773–16610	0	

*H and L refer to the heavy and light strand, respectively.

### Transfer RNA genes and ribosomal RNA genes

**Figure 4:**
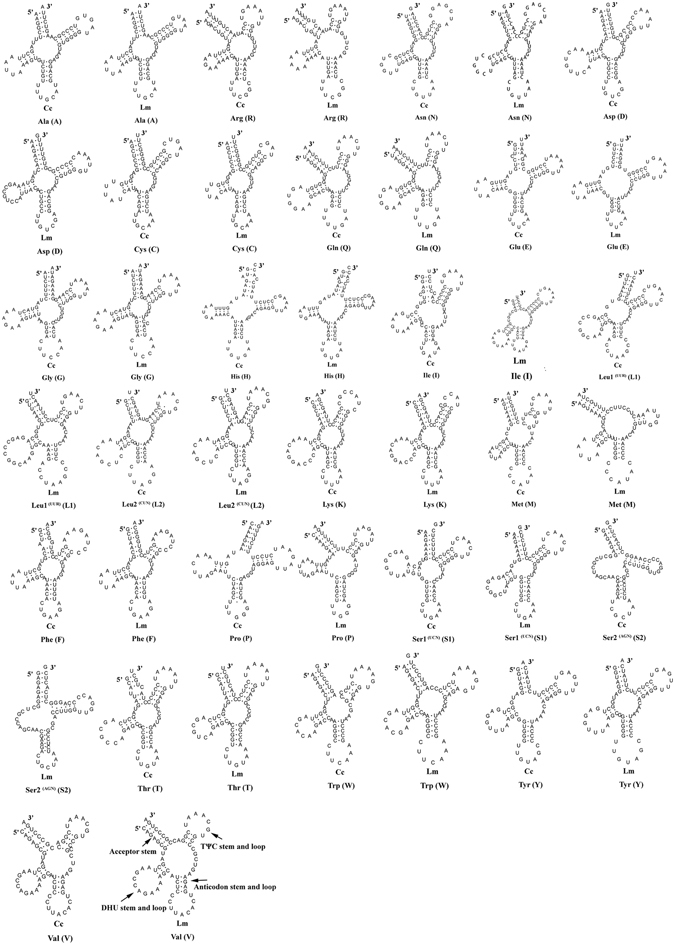
Predicted secondary structures for the tRNA genes in the *C. kumu* and *L. microptera* mitogenome.

The predict result of 22 tRNA genes (two for Serine and Leucine and one for each of the other amino acids) in the two fish mitogenomes are showed from Fig. 4. Fourteen tRNAs are encoded by the H-strand, and the remaining 8 tRNAs are encoded by the L-strand (Table 2). All tRNAs varied in size from 62 bp (trnC) to 74 bp (trnL1 (UUR) and trnK) in *C. kumu* and ranged from 65 bp (trnC) to 74 bp (trnL1 (UUR) and trnK) in *L. microptera*. This tRNA genomic architecture is identical to all Scorpaeniformes species examined to date^39, 40^. All of the 22 tRNA genes could be folded into typical cloverleaf secondary structures with the exception of trnS2 (AUN). The secondary structure of trnS2 (AUN) lacks a DHU stem, and this phenomenon has been observed in many animals of metazoan mitogenomes including Scorpaeniformes species^39, 40^. A 7 bp amino acid acceptor stem is conserved in all tRNAs, except for trnaV and trnaL1 (UUR) in *L. microptera*, which has a 9 bp amino acid acceptor stem. Additionally, 28 to 33 unmatched base pairs are found in the secondary structure of all tRNAs in both fish mitogenomes. All of the unmatched base pairs are G-U pairs, which formed a weak bond, except for the U-U pair in the TΨC stem of Leu2 in *C. kumu*. A positive AT skew (0.1127 and 0.1193) and a negative GC skew (−0.1011 and −0.1067) are found among the concatenated sequence of all 22 tRNAs in *C*. *kumu* and *L*. *microptera* respectively, indicating A nucleotides are more likely to occur than Ts, so are Cs nucleotides than Gs. A similar result occurred in the ribosomal genes in both species. In mitogenomes of *C*. *kumu*, the 12S and 16S rRNA genes are 946 bp (51.0% A + T content) and 1653 bp (53.1% A + T content), respectively. As in mitogenomes of *L. microptera*, the length of 12S rRNA is 1041 bp with an A + T content of 51.2% and the 16S RNA is 1695 bp with an A + T content of 53.2%. The two rRNAs are located between trnF and trnL1 (UUR). They are separated by the trnV. The location was the case in most vertebrates^34, 35, 39, 40^.

### Non-coding regions

**Figure 5:**
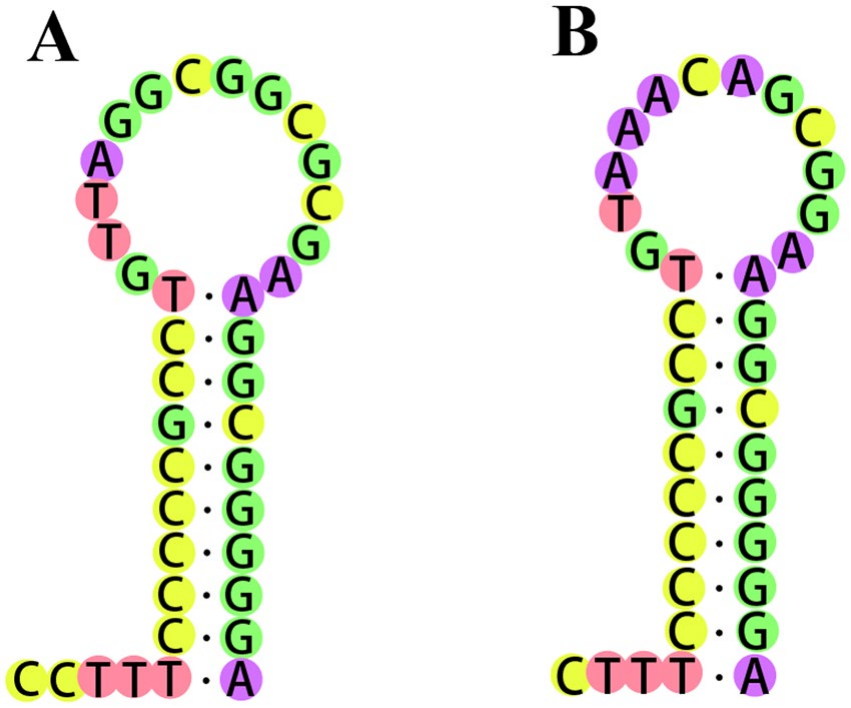
The putative hairpin secondary structure of the O_L_ found in the *C. kumu* (**A**) and *L. microptera* (**B**) mitogenome.

**Figure 6:**
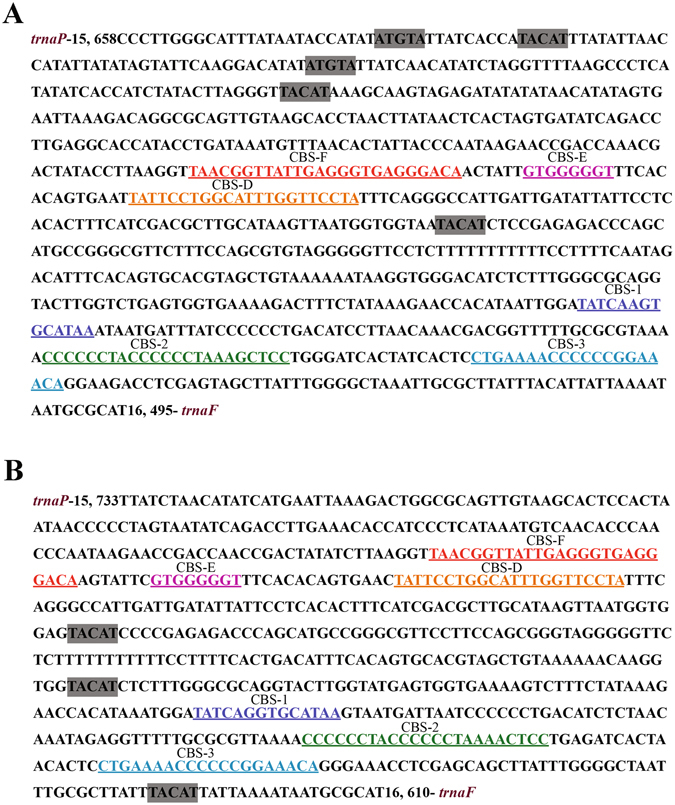
Features present in the control regions of the *C. kumu* (**A**) and *L. microptera* (**B**) mitogenomes. The conserved sequence blocks contain CSB-F (red), CSB-E (purple), CSB-D (orange), CSB-1 (deep blue), CSB-2 (green) and CSB-3 (light blue). The conserved motifs ATGTA and its complement TACAT which may form thermostable hairpin structure are shaded.

Except for the several short non-coding regions between genes, there are also two long non-coding regions in the mitochondrial genomes of *C. kumu* and *L. microptera*: O_L_ and CR, which are important in the transcription, replication and maintenance of the mitochondrial genome^41^. In mitogenomes of *C. kumu* and *L. microptera*, O_L_ is found in the domain between trnN and trnC, which is folded a hairpin secondary structure (Fig. 5). The length of *C. kumu* is 36 bp in *C. kumu* and 38 bp in *L. microptera*, respectively. The control region is essential for the initiation of replication in vertebrates, which is found between tRNA^Pro^ and tRNA^Phe^. The CR in *C. kumu* spanned 838 bp with a 60.8% A + T content and shows an equal AT skew (0) and a negative GC skew (−0.0793). The CR in *L. microptera* spanned 838 bp with 59.3% A + T content and shows a slightly positive AT skew (0.0181) and a negative GC skew (−0.1144). The A + T content of the two mitogenomes is lower than most of the other Scorpaeniformes species to date^33, 34^. Several conserved sequence blocks (CSBs) are observed in the control regions of teleost fish, which could play important roles in mitochondrial metabolism^42^. In CR of teleost fish, CSB-D, -E and -F are found in central conserved domains. Meanwhile, CSB-1, CSB-2 and CSB-3 are typically present in the conserved sequence block domain^42^. All the CSBs are identified in *C. kumu* and *L. microptera*, by comparing them with the recognition sites in Scorpaeniformes species (Fig. 6). Both species have no relatively similar repetitive motifs in the control regions, which indicated that the repeat motifs occurred after family diversification. However, more information is necessary to further evaluate evolutionary aspects of repeat clusters. In addition, tandem repeats are not found in the two species using the Tandem Repeats Finder program^25^. This phenomenon exists for other Scorpaeniformes species with the exception of the genus *Sebastes* ^33, 34^.

### Phylogenetic analysis

**Figure 7:**
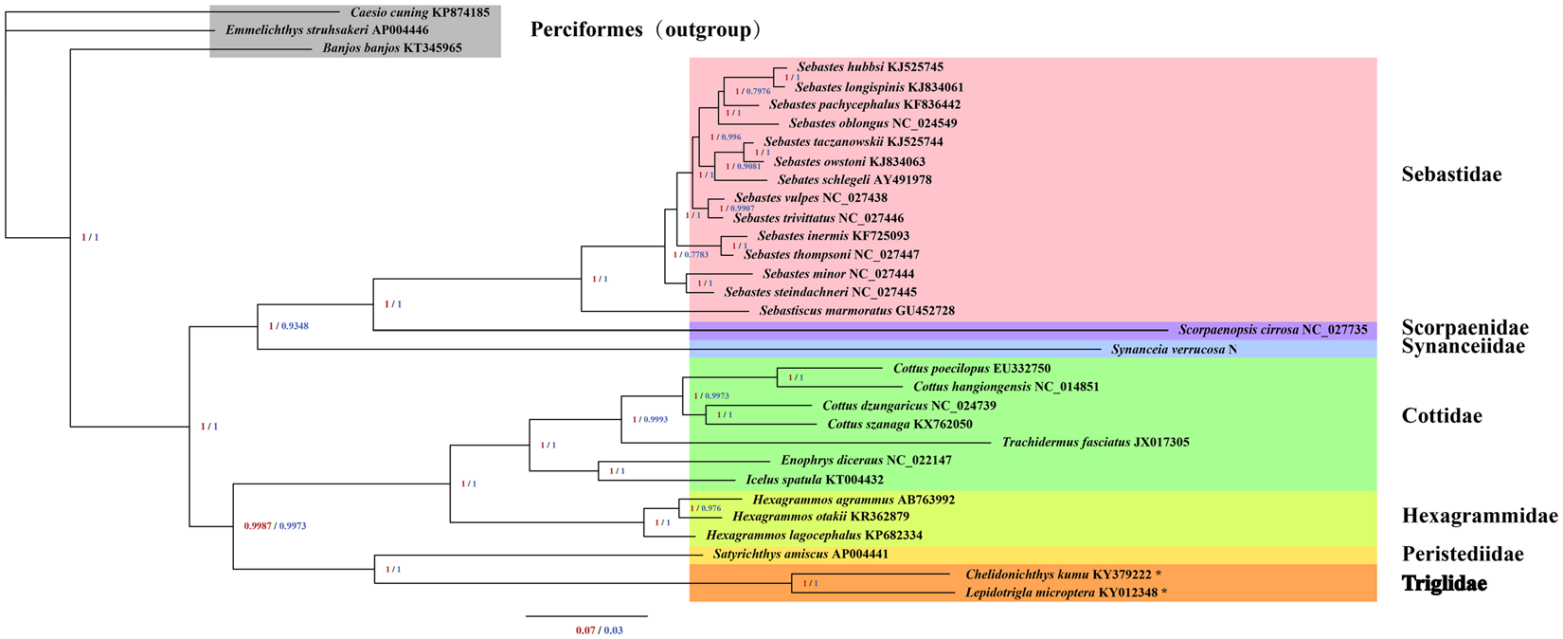
Phylogenetic trees inferred from amino acid and nucleotide sequences of 13 PCGs of the mitogenome using BI analysis. Perciformes fish (*C. cuning*, *E. struhsakeri* and *B. banjos*) were used as outgroups. The numbers along branches indicate posterior probability values (red for amino acid and blue for nucleotide sequences). The accession numbers are shown behind the species names.

The interrelationship of Scorpaeniformes remains contentious due to the lack of corroborative evidence^43^. In this study, six Scorpaeniformes families of the 13 PCGs from mitogenomes, concatenated nucleotide sequences and amino acids, are used to reconstruct phylogenetic relationships via the BI and ML methods (Figs 7 and S1). The results provide good support for the monophyly of each family. The phylogenetic trees include 29 Scorpaeniformes species, representing 7 families and 11 genera. The best supported phylogenetic relationship found in this study is as follows: (Synanceiidae + (Scorpaenidae + Sebastidae)) + ((Peristediidae + Triglidae) + (Cottidae + Hexagrammidae)). We sequenced two species (*C. kumu* and *L. microptera*) within Triglidae, which formed a monophyletic group with Peristediidae. The analysis suggests that Peristediidae are most closely related to Triglidae. The topology of the relationships of Scorpaeniformes is similar to previous work^13^, and more mitogenomes from Triglidae fish are needed for further phylogenetic analyses and to demonstrate the relationships among these families in the future.

## Conclusion

The first two complete mitochondrial genomes (*C. kumu* and *L. microptera*) for the family Triglidae were determined and compared with those of other Scorpaeniformes species. The mitogenome sequences of *C. kumu* and *L. microptera* were 16,495 bp and 16,610 bp in length, respectively. Each mitogenome is composed by the typical structure of 13 PCGs, 2 rRNAs, 22 transfer RNA genes and two non-coding regions. Both of the complete mitogenomes of Triglidae fish were typical circular molecules and had similar genome organization and structure as those found in other teleostean species. Similar to other vertebrate mitogenomes, most of the PCGs utilized ATG, except for *cox1*. Additionally, all of the tRNAs could be folded into typical cloverleaf secondary structures with the exception of trnS2 (AUN), which lacks a DHU stem. Several CSBs were found in the control regions of the two fish, which could play important roles in mitochondrial metabolism. By phylogenetic analysis, the mitogenome sequences could be used to resolve the higher-level relationship of Scorpaeniformes. The results showed that Triglidae are most closely related to Peristediidae. Our findings provided important data for further studies on the population genetics and evolutionary biology of Scorpaeniformes. Additional taxonomic work is needed in the future.

## Electronic supplementary material

Supplementary Information
