# Supplementary material for: The first two complete mitochondrial genomes for the family Triglidae and implications for the higher phylogeny of Scorpaeniformes
Source: Sci Rep. 2017 May 8;7:1553. doi: 10.1038/s41598-017-01654-y (PMC5431562; doi:10.1038/s41598-017-01654-y)
Supplement: Supplementary file 1 — Supplementary Information [file 41598_2017_1654_MOESM1_ESM.pdf]

The first two complete mitochondrial genomes for the family Triglidae and implications for the higher phylogeny of Scorpaeniformes

Lei Cui[1], Yuelei Dong[1], Fenghua Liu[1], Xingchen Gao[2], Hua Zhang[1], Li Li[1], Jingyi Cen[1] & Songhui Lu[1*]

[1] Key Laboratory of Eutrophication and Red Tide Prevention, Research Center for Harmful Algae and Marine Biology, Jinan University, Guangzhou 510632, China

[2]Chinese Sturgeon Research Institute, Three Gorges Corporation, Yichang, 443100, China

Primer	Sequences(*C. kumu*)	Length (bp)	Annealing temperature(℃)
1F	CCATGCCTTCGTAATGATTTTC	2818	55
1R	TCGTCAGCGGAGACTAGCAC		
2F	ACTCATACATAATCCCCACCCA	2601	55
2R	GGTGCTCGGATGTAAAGTGGTA		
3F	ATGGAGGGAGAACGAAAACAA	3587	55
3R	GAGGCAGTTTGGTTGGTTTCTA		
4F	AGCCCAATTTGGACTTCACC	2569	55
4R	GGAAGATGAGGTGGATAAGGGT		
5F	TCGAGATGTGAACTACGGATGG	3618	55
5R	GAAGAAAGAAGCCGGGAGG		
6F	GTGGCAAAAGAGTGGGAAGAG	2401	55
6R	TGGGAGTGTTGGGATGTGG		
7F	CGCTGGCAGAAACTAACCG	2876	55
7R	TGGTGTTTAGGTTGCGGTCA		
Primer	Sequences(*L. microptera*)	Length (bp)	Annealing temperature(℃)
1F	TGACAGTCACCGGCGTAAAG	3229	55
1R	AGGTGGGCAATAGAAGAGTAAGC		
2F	TAACAGCTAAACGCTCAAACCA	1904	55
2R	TCCTAGTTGTGACGGATGTGC		
3F	ATCTTCTCCCGTCCTATTCCTG	2254	55
3R	GCAAGGAGGAGAAGGAAAGATG		
4F	CTAATCACAAAGACATTGGCACC	4518	55
4R	GAAGGCTGATGAGAATGCAAAG		
5F	TCTTAGTAATAGCAACTGCCTTCG	2160	55
5R	GGTTCAGGGAAGATAAAGCCAC		
6F	CTGAGGGTGAGTCGCCTGTA	3398	55
6R	AGTATCATTCGGGCTTGATGTG		

Table S1 Primer pairs used for PCR amplification of *C. kumu* and *L. microptera* mitogenomes.

Codon	n(RSCU)		Codon	n(RSCU)		Codon	n(RSCU)		Codon	n(RSCU)	
	C. kumu	*L. microptera*		*C. kumu*	*L. microptera*		*C. kumu*	*L. microptera*		*C. kumu*	*L. microptera*
UUU(F)	105(0.91)	69(0.73)	UCU(S2)	53(1.31)	47(1.25)	UAU(Y)	26(0.47)	24(0.49)	UGU(C)	11(0.92)	9(0.75)
UUC(F)	127(1.09)	120(1.27)	UCC(S2)	79(1.96)	69(1.83)	UAC(Y)	84(1.53)	74(1.51)	UGC(C)	13(1.08)	15(1.25)
UUA(L2)	59(0.54)	41(0.42)	UCA(S2)	44(1.09)	37(0.98)	UAA(*)	8(3.56)	12(1.55)	UGA(W)	97(1.64)	87(1.57)
UUG(L2)	14(0.13)	11(0.11)	UCG(S2)	9(0.22)	7(0.19)	UAG(*)	1(0.44)	3(0.39)	UGG(W)	21(0.36)	24(0.43)

CUU(L1)	150(1.37)	143(1.46)	CCU(P)	57(1)	46(0.81)	CAU(H)	26(0.48)	21(0.4)	CGU(R)	12(0.61)	16(0.78)
CUC(L1)	203(1.85)	188(1.92)	CCC(P)	120(2.11)	126(2.22)	CAC(H)	83(1.52)	83(1.6)	CGC(R)	24(1.22)	27(1.32)
CUA(L1)	179(1.63)	151(1.54)	CCA(P)	37(0.65)	42(0.74)	CAA(Q)	87(1.78)	87(1.64)	CGA(R)	37(1.87)	30(1.46)
CUG(L1)	54(0.49)	53(0.54)	CCG(P)	13(0.23)	13(0.23)	CAG(Q)	11(0.22)	19(0.36)	CGG(R)	6(0.3)	9(0.44)
AUU(I)	118(0.9)	104(0.91)	ACU(T)	48(0.63)	47(0.64)	AAU(N)	21(0.37)	32(0.6)	AGU(S1)	10(0.25)	11(0.29)
AUC(I)	145(1.1)	125(1.09)	ACC(T)	140(1.85)	132(1.8)	AAC(N)	94(1.63)	74(1.4)	AGC(S1)	47(1.17)	55(1.46)
AUA(M)	77(1.05)	69(1.08)	ACA(T)	105(1.39)	100(1.36)	AAA(K)	60(1.67)	63(1.68)	AGA(S1)	0(0)	11(1.42)
AUG(M)	70(0.95)	59(0.92)	ACG(T)	10(0.13)	15(0.2)	AAG(K)	12(0.33)	12(0.32)	AGG(S1)	0(0)	5(0.65)
GUU(V)	53(0.91)	43(0.98)	GCU(A)	55(0.63)	48(0.65)	GAU(D)	20(0.53)	14(0.44)	GGU(G)	33(0.55)	24(0.49)
GUC(V)	89(1.53)	53(1.2)	GCC(A)	189(2.15)	153(2.06)	GAC(D)	55(1.47)	49(1.56)	GGC(G)	98(1.62)	94(1.94)
GUA(V)	57(0.98)	58(1.32)	GCA(A)	90(1.03)	87(1.17)	GAA(E)	75(1.49)	67(1.6)	GGA(G)	58(0.96)	45(0.93)
GUG(V)	33(0.57)	22(0.5)	GCG(A)	17(0.19)	9(0.12)	GAG(E)	26(0.51)	17(0.4)	GGG(G)	53(0.88)	31(0.64)

Table S2. Codon number and RSCU in *C. kumu* and *L. microptera* mitochondrial PCGs. A total of 3,808 and 3,431codons were analysed respectively, excluding the initiation and termination codons. Amino acids encoded by these codons are labelled according to the IUPAC-IUB single-letter amino acid codes.

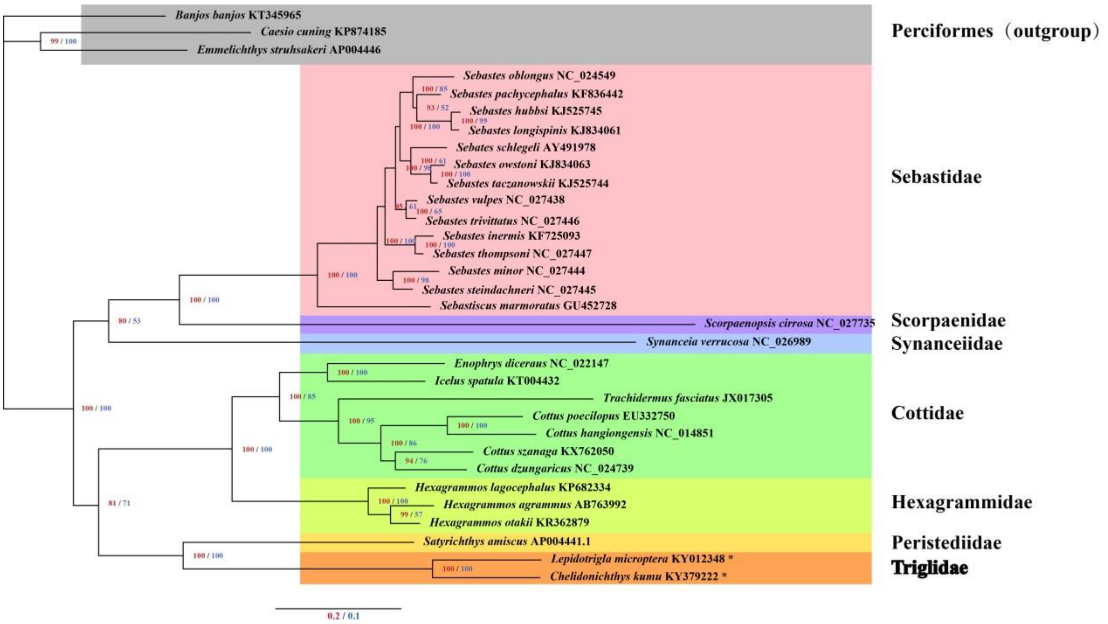

Figure S1. Phylogenetic trees inferred from amino acid and nucleotide sequences of 13 PCGs of the mitogenome using ML analysis. Perciformes fish (*C. cuning*, *E. struhsakeri* and *B. banjos*) were used as outgroups. The numbers along branches indicate posterior probability values (red for amino acid and blue for nucleotide). The accession numbers are shown behind the species names.

Accession codes: The *C. kumu* and *L. microptera* mitogenomes were submitted under the accession number KY379222 and KY012348 to NCBI, respectively.

Competing financial interests: The authors declare no competing financial interests.
